# RAEN: Rate Adaptation for Effective Nodes in Backscatter Networks

**DOI:** 10.3390/s22124322

**Published:** 2022-06-07

**Authors:** Jumin Zhao, Qi Liu, Dengao Li, Qiang Wang, Ruiqin Bai

**Affiliations:** 1 College of Information and Computer, Taiyuan University of Technology, Taiyuan 030024, China; lq15735151998@163.com (Q.L.); wangqiang510@126.com (Q.W.); bairuiqinty@163.com (R.B.); 2 Key Laboratory of Big Data Fusion Analysis and Application of Shanxi Province, Taiyuan 030024, China; lidengao@tyut.edu.cn; 3 Intelligent Perception Engineering Technology Center of Shanxi, Taiyuan 030024, China; 4 College of Data Science, Taiyuan University of Technology, Taiyuan 030024, China

**Keywords:** rate adaptation, effective nodes, backscatter networks, Gaussian model

## Abstract

A backscatter network, as a key enabling technology for interconnecting plentiful IoT sensing devices, can be applicable to a variety of interesting applications, e.g., wireless sensing and motion tracking. In these scenarios, the vital information-carrying effective nodes always suffer from an extremely low individual reading rate, which results from both unpredictable channel conditions and intense competition from other nodes. In this paper, we propose a rate-adaptation algorithm for effective nodes (RAEN), to improve the throughput of effective nodes, by allowing them to transmit exclusively and work in an appropriate data rate. RAEN works in two stages: (1) RAEN exclusively extracts effective nodes with an identification module and selection module; (2) then, RAEN leverages a trigger mechanism, combined with a random forest-based classifier, to predict the overall optimal rate. As RAEN is fully compatible with the EPC C1G2 standard, we implement the experiment through a commercial reader and multiple RFID tags. Comprehensive experiments show that RAEN improves the throughput of effective nodes by 3×, when 1/6 of the nodes are effective, compared with normal reading. What is more, the throughput of RAEN is better than traditional rate-adaptation methods.

## 1. Introduction

Internet of things (IoT) systems require a universal and user-oriented perceptual recognition layer, to sense and collect a great amount of data from humans, objects, and the environment. Backscatter networks [1] that can realize non-optical proximity, low-power transmission, and multiple-node inventory just fit this requirement. Using radio waves, the battery-free backscatter nodes—such as RFID tags—can help sensors realize wireless charging and connection [2,3]. As the market scale of the IoT continues to expand, when the node has a large amount of data to transmit or when it is in a fast-moving state, the reader needs to collect its information in a timely and effective manner [4].

To expand application scenarios, it is very important to improve the throughput of backscatter networks. Among them, the rate-adaptive (RA) algorithm has attracted wide attention, due to its advantages of improving the anti-jamming ability of a link without hardware modification [5,6,7,8]. The RA algorithm adjusts the transmission rate and encoding mode of backscatter nodes, according to the changes in the external environment, so as to improve the transmission success rate of the message.

Blink [5] is the first scheme that applies the rate-adaptation algorithm to a backscatter system, but it only considered the frequency diversity of one node. To address it, CARA [6] exploits both spatial and frequency diversity, to maximize the network throughput. Focusing on the mobile scenario, MobiRate [7] introduces a velocity-based loss-rate-estimation method and improves the accuracy rate of rate selection. While prior works have left the downlink unattended, RAB [8] chooses the best rate for both the downlink and uplink.

However, the existing RA algorithms provide the same transmission opportunities for all nodes with a lack of consideration for differences between nodes. When a large number of nodes exist, such systems are extremely prone to collisions. Especially when the system resources are limited, the transmission of invalid information will only occupy a limited time slot. For example, in practical applications, managing numerous tagged objects requires fast tracking their frequent rearrangement [9,10,11]. The movement of the tagged object will cause the information of the corresponding tag to change. In this scenario, mobile nodes that can provide useful information deserve attention, while the stationary nodes are the opposite. Taking environmental monitoring [12,13,14] as another example, when a large number of nodes are used to collect environmental information in factories, forests, and coal mines, useful information, often, comes from the node with a channel that is dynamic. In order to have a higher detection accuracy, it is necessary to improve the sampling rate of nodes, so a fast information-collection method is very important.

In both cases, we refer to the backscatter nodes that carry useful information as effective nodes. If not, they are called invalid nodes. According to the above analysis, the effective nodes can be divided into two categories: (1) a moving node and (2) a static node, with a channel that is dynamic.

In this paper, to improve the wireless transmission capability of effective nodes, we propose to consider the efficiency diversity between backscatter nodes and extract effective nodes for exclusive rate optimization. In order to design an algorithm that is suitable for effective nodes, there are three challenges to overcome:

(1) Exclusively extracting the effective nodes is challenging. We need to design methods to exclusively identify and select effective nodes. However, it is difficult to identify effective nodes, since the movements of effective nodes are random, and their communication environment is changing rapidly. Besides, considering the huge number of nodes, simply using the one-by-one method is not feasible.

(2) Designing a proper algorithmic trigger is challenging. According to the framework of the existing RA algorithm, a trigger is required to determine the start time of rate optimization. However, these triggers need to monitor channel mobility, continuously, and introduce additional overhead. So, how to redesign the trigger is a problem.

(3) Making reasonable rate decisions is challenging. According to the EPC C1G2 standard [15], the RFID reader communicates with all backscatter nodes at the same rate. Although we have predicted an optimal transmission rate for every node, only one rate can be chosen in the end. Gong et al. [6] propose to choose the highest data rate, among all nodes’s optimal data rates, as the final rate. However, this method lacks the consideration of fairness, and adds insult to injury for the nodes with a transmission rate that is low. So, a fairer rate-decision method is needed.

According to the above challenges, we present an RA algorithm that is suitable for effective nodes, called RAEN, to improve the effective throughput of the system. The contributions of this study are as follows:

(1) Compared with existing works, RAEN is the first algorithm that considers the efficiency diversity between backscatter nodes. In our design, we exclusively extract effective nodes through a renewable Gaussian model (GM) and a selection mechanism, then, we adopt the rate-optimization method to further improve their throughput.

(2) We design a rate-optimization algorithm for effective nodes, in which a new trigger and a rate-decision module are designed. The trigger can save detection overhead, without monitoring the change of external environment. The rate-decision module takes the Received Signal Strength Indicator (RSSI) and packet-loss rate as indicators and determines the overall network rate based on a random forest method. By this method, the optimal rate is decided more fairly, and the reading rates of effective nodes are significantly increased.

(3) We evaluate our design through a trace-driven simulation, as the experimental results show that RAEN improves the goodput of effective nodes by 3×, when 1/6 of nodes are effective.

Other parts are as follows: Section 2 describes the overview of RAEN. Section 3 shows the method of effective node extraction. In Section 4, the method of rate optimization is analyzed. Section 5 is the implementation and evaluation part. Section 6 is the discussion. Finally, the conclusion is given.

## 2. Overview

**Figure 1:**
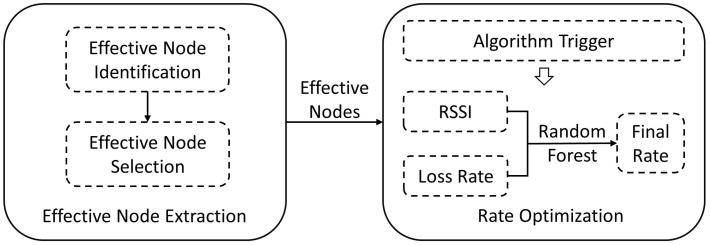
System overview.

RAEN aims to adaptively change the data rate of effective nodes. As shown in Figure 1, it mainly includes two stages: effective node extraction and rate optimization.

In the first stage, RAEN aims to extract effective nodes exclusively. First, an identification part is adopted to find the effective node, according to its phase information. This is done by judging whether the phase of the node follows a renewable Gaussian model, which is built by previous phases. Second, a selection part is adopted to uniformly isolate the effective nodes from others, with a lesser SELECT command, by assigning each node a bit vector.

In the second stage, RAEN leverages a trigger mechanism, combined with a random forest classifier, to predict the overall optimal rate. The trigger mechanism does not detect the change of the channel, only needing to count the inventory cycle to determine the time of the changing rate. When changing rate, RAEN leverages a random forest classifier to map two indicators (RSSI and packet loss rate), to an appropriate data rate.

## 3. Effective Nodes Extraction

### 3.1. Effective Nodes Identification

According to the description in Section 1, effective nodes that carry the most valuable information are caused by two reasons. One is the moving of the node, another is the change of the channel. These two reasons will cause changes in phase and RSSI. In this paper, we use the Gaussian model to detect the jitter of the phase, to identify effective nodes. The reason we use phase, instead of RSSI, for identification is that its quantization is more precise and, thus, more sensitive to changes [16].

#### 3.1.1. Gaussian Model

**Figure 2:**
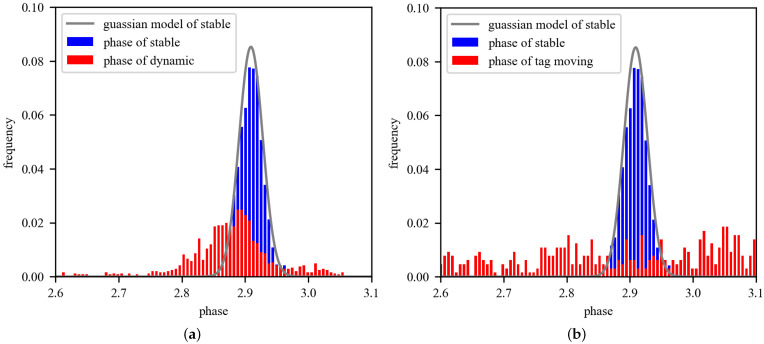
Gaussian distribution of RF phase in different scenarios. (**a**) Description of the change of phase distribution, when the environment becomes dynamic. (**b**) Description of the change of phase distribution, when the node is moving.

Due to the interference of Gaussian White Noise, introduced by the thermal motion of electrons, the RF phase in a wireless communication network obeys a typical Gaussian distribution, instead of an accurate value [17]. A backscatter network is not exceptional. To verify this, we placed a node 1 m away from the reader’s antenna and measured its phase values for 10 s. We, first, obtained the phases of the stationary node under a stable environment and calculated the occurrence probability of each value, as shown in the blue bar of Figure 2. Apparently, the phase value of the stationary node follows a Gaussian distribution. Moreover, we designed two experiments to analyze the influence of the environment changing and the node moving: (1) we had volunteers walking around the nodes to create a dynamic environment, and (2) we make the nodes move at a constant speed, of about 0.5 m/s, perpendicular to the antenna. In both cases, the phase of the node no longer follows the previous distribution, as shown in the red bar of Figure 2.

Based on the above analysis, we put forward the method of detecting effective nodes. Assuming that the current RF phase values of a particular node are $\theta_{1}$, $\theta_{2}$, …, $\theta_{n}$. If the node is stable, these phases will follow a Gaussian distribution. As for the incoming new phase $\theta_{n+1}$, its value will follow the previous Gaussian model, i.e.,

(1) $$\theta_{n+1}∼n\left(\mu_{n},\delta_{n}\right),$$

where $\mu_{n}$ and $\sigma_{n}$ are the expectation and the standard deviation of the history phase values, respectively. Accordingly, the probability density function of $\theta_{n+1}$ is given by:

(2) $$\eta \left(\theta_{n+1},\mu_{n},\delta_{n}\right)=\frac{1}{\delta_{n}\sqrt{2\pi }}\exp\left(-\frac{{\left(\theta_{n+1}-\mu_{n}\right)}^{2}}{2\sigma_{n}^{2}}\right).$$

Thus, we can utilize this characteristic to distinguish whether the node is effective or not. We assume the node is effective, if the incoming phase value does not match the Gaussian model, i.e., $\left|\theta_{n+1}-\mu_{n}\right|/\delta_{n}>\xi$, where ξ is a user-defined parameter, to describe the matching degree of the phase to the model. The larger $\left|\theta_{n+1}-\mu_{n}\right|/\delta_{n}$ is, the more effective the node is, and vice versa.

#### 3.1.2. Update of Gaussian Model

Before identification, the reader should establish an initial Gaussian model for each node, through several inventory cycles, and the Gaussian model should be updated, subsequently. For the current phase value, we will update the mean and standard deviation of the model, based on this new value. To achieve this, we borrow the method from computer vision [18], as follows:

(3) $$\left\{\begin{array}{c} \mu_{k,n+1}=\left(1-\rho \right)\mu_{k,n}+\rho \theta_{n+1}\\ \delta_{k,n+1}=\sqrt{\left(1-\rho \right)\delta_{k,n}^{2}+\rho {\left(\theta_{n+1}-\mu_{n+1}\right)}^{2}} \end{array}\right.$$

where ρ is equal to α multiplied by $\eta \left(\theta_{n+1},\mu_{n},\delta_{n}\right)$, α is a learning rate, and $\eta \left(\theta_{n+1},\mu_{n},\delta_{n}\right)$ is the probability of $\theta_{n+1}$, defined in (3). According to our experiments, the learning rate of the model is positively correlated with the level of the efficiency of the node. To deal with this issue, the value of α is set to 0.002, if the node is effective; otherwise, it is set to 0.001.

### 3.2. Effective Nodes Selection

#### 3.2.1. The SELECT Command

The SELECT command allows a reader to select the inventory of a node subset, enabling node partitioning based on union (∪), intersection (∩), and negation (∼). So, this command is the core part of extracting the effective nodes. The command implements its functions with six fields: Target, Action, Membank, Pointer, Length, and Mask.

**Target, Action**: The Target field indicates whether a SELECT modifies a node’s selected flag (SL) or inventoried flag. In conjunction with Target field, the Action field determines how to set the chosen flag. Combining this two fields, the reader is able to modify the specific flag of a group of backscatter nodes, so as to operate the nodes with the desired flag value. The criteria for determining whether a node is matched or not is specified by the Membank, Pointer, Length, and Mask fields.

**Figure 3:**
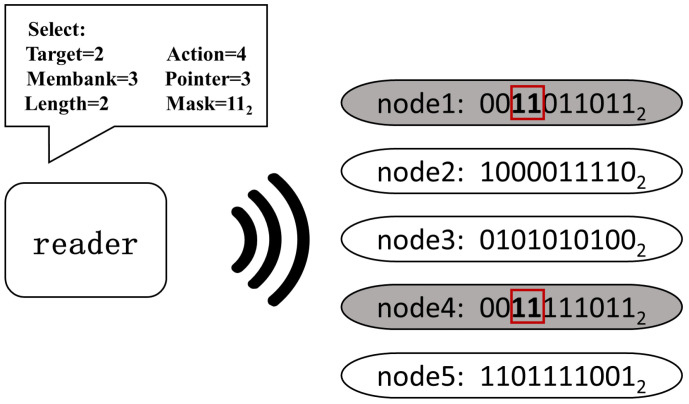
An example of SELECT.

**Membank, Pointer, Length, Mask**: A memory area is located according to these four fields. Membank specifies a memory bank for the area, where the value of 0, 1, 2, and 3, respectively, indicates the Mask is applied to the Reserved memory, EPC memory, TID memory, and User memory. Pointer field and Length field, respectively, indicate the starting position and length of the memory area. After the memory area has been chosen, it will be compared with the Mask fields. If the same, the corresponding node is matching, and vice versa. As shown in Figure 3, there are a total of five nodes, in the field of view. For efficiency, in the following text, *S* (*t*, *a*, *b, p*, *l*, or *m*) is specified to denote the SELECT command, where *t*, *a*, *b*, *p*, *l*, and *m*, respectively, indicate Target, Action, Membank, Pointer, Length, and Mask. Each node has a bit vector in the same memory location. To select node1 and node4, it can be observed that the third and fourth bits in their segments are “1”, while the other nodes are “0”. By a specific SELECT command S (2, 4, 3, 3, 2, or 11), node1 and node4 are selected, while the others are left.

Based on the above analysis, it can be concluded that the area to be matched has a significant influence on the selection result. If a specific node subset is wanted, the simplest approach is to select its unique EPC codes. However, this method is inefficient, since one SELECT command can only pick one node. This cannot meet the needs of practical applications, such as large-scale backscatter networks, where numerous masks are needed to collect nodes. So, how to improve the efficiency of selection is an important problem.

#### 3.2.2. The Bit-Vector Method

According to the above analysis, the method of matching the exclusive EPC code to the mask is very inefficient, since N nodes require N SELECT commands. Considering the real-time performance of the scheme, we want to use very few SELECT commands, or even one, to match multiple effective nodes. Inspired by the method called WB [19], a specific bit vector is used to encode the node. Given a node set $\Gamma =\left\{t_{1},t_{2},\ldots t_{n}\right\}$, an area of 2*n* bits long is specialized. We set the EPC code of the node from 1 to *n*. For the ith node $t_{i}$, with an EPC code that is *i*, the *i*th bit and the (n+i)th bit of the bit vector are set to 1; the rest are set to 0. To pick a consecutive node set $\Gamma_{ij}=\left\{t_{i},t_{i+1},\ldots t_{j}\right\}$, we just need to check if the bit vector, which started from the (j+1)-th bit and moved to the (n+i−1)-th bit, is equal to 0.

**Figure 4:**
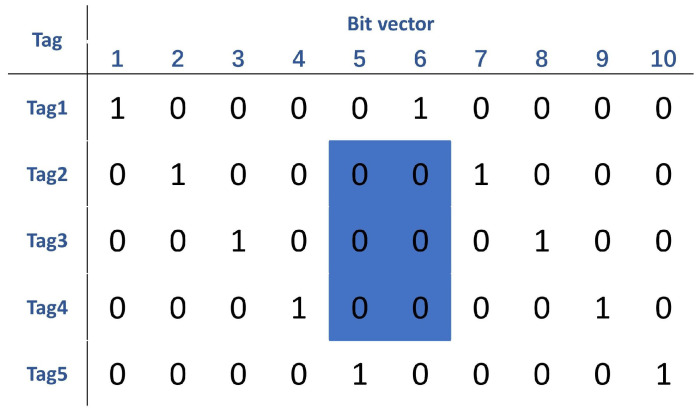
Bit vectors and the selection of node2, node3, and node4.

By issuing a command, which is denoted by $S\;(t,a,b,j+1,n+i-j-1,00_{2})$, the matched nodes will be picked. As shown in Figure 4, the node set $\Gamma =\{t_{1},t_{2},t_{3},t_{4},t_{5}\}$, with a bit vector that is 10 bits long, is considered. To select $t_{2}$, $t_{3}$, and $t_{4}$, a mask $00_{2}$ that starts at the 5th bit and ends at the 6th bit can, uniformly, isolate these nodes from others. Accordingly, the SELECT command to pick this set is: $S\;(t,a,b,5,2,00_{2})$. To design a selection method that fits our scheme, the values of each field are discussed.

**The value of “*****t*****”**: It is important to decide whether the selected flag or inventoried flag is used for the subsequent inventory. If choosing to label the inventoried flag, the node will invert its inventoried flag (i.e., A→B or B→A), when it receives a Query message that will not meet the requirements of multi-node inventory. However, this is not going to happen, if choosing to label the selected flag, since a node only refreshes its selected flag when it receives a SELECT message or when it is powered up. In this manner, “*t*” is set to 4.

**The value of “*****a*****”**: Although our algorithm allows one SELECT command to pick multiple nodes with continuous coding, in a real scenario, multiple commands are required to pick all effective nodes. Under the circumstances, “*a*” is set to 1, so new matching nodes are picked up and non-matching nodes do nothing.

**The value of “*****b*****”**: Considering that only the User memory bank allows users to make modifications, “*b*” is set to 3.

Based on the above analysis, we can conclude the process of extracting the effective node set. First, if the reader detects that the phase of the node does not match the previous Gaussian model, it will record the EPC codes of these nodes. Second, the reader divides these nodes into many consecutive groups, according to their EPC codes, then, it issues corresponding SELECT commands to pick up all the effective nodes.

## 4. Rate Optimization

**Figure 5:**
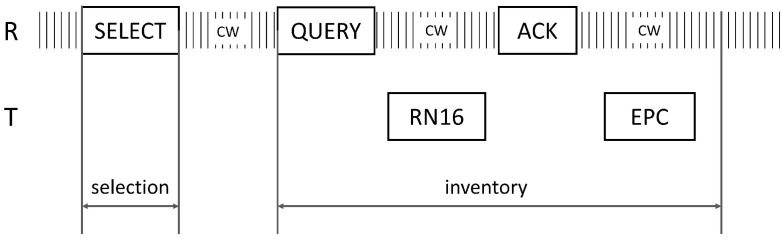
Selection and Inventory stages, in EPC C1G2.

When optimizing the rate, it is necessary to understand the communication protocol between the reader and the tag. According to the EPC C1G2 protocol, the operation from reader to node is divided into three stages: Selection, Inventory, and Access. Usually, for a new passive backscatter node that has integrated ultra-low power sensors, EPC is a better choice for loading sensor data, in terms of control overhead and slot efficiency [19]. Therefore, we will only consider the Selection and Inventory stages in the following text, as shown in Figure 5.

### 4.1. Trigger

When we implement rate optimization, the first step is to decide the start time of the algorithm. While many prior efforts have tried to solve this, they all need additional calculation or probe packets, to form an algorithm trigger. For example, Blink [5] calculates the distance between RSSI vectors and loss rate vectors, to determine mobility; Cara [6] utilizes the Bhattacharyya distance between two histograms of phase differences over a short interval, to determine whether to change the rate. In this paper, we design a new algorithm trigger that is related to the inventory cycle, to save the detection overhead.

In terms of sensor nodes, with transmission data that are loaded in EPC code, the Inventory stage, which contains a sequence of reader commands and node responses, is inevitable. Commands and responses that must be carried out include:

**Query, Query Rep, Query Adj**: A Query message initiates a communication round and formulates the rate mode of the current inventory cycle. A critical parameter specified by Query is *Q*. It defines the number of slots in each inventory cycle. According to FCC regulation, in each inventory cycle, readers can only use one fixed-rate mode; the function of Query just fit this regulation. This inspired us to associate the inventory cycle with the rate-adaptive trigger. Except for Query, a Query Req message can start a rest slot with less cost, and a Query Adj message can be up to this task, by adjusting the current *Q* value within 1 step.

**ACK (RN16)**: When the slot counter reaches 0, the node will send a 16-bit random RN16 to the reader. Upon decoding the RN16, the reader will echo the RN16 it received as an ACK.

**EPC**: A node knows it is time to send an EPC code, if the received ACK matches the sent RN16. After sending its EPC, the node will not respond to subsequent Query Reps or Query Adjusts, during this round of inventory.

However, considering the double function of Query, we try to trigger the algorithm from the perspective of inventory cycle. Specifically, this trigger mechanism aims to calculate the number of inventory cycle. Once the number reaches a predefined threshold (denoted by *M*), the reader will switch the rate mode. Since commercial readers do not, directly, expose the relevant parameters, we calculate the number of inventory cycle according to the maximum read number of all nodes. Considering the accuracy and efficiency of the algorithm, the selection of *M* must be appropriate. A small M means the reader has to change the rate very frequently, which will introduce a delay of tens of milliseconds, each time the reader switches the rate. A big *M* can hardly improve throughput because rate changes cannot keep pace with the state changes. The value of *M* will be determined later. After that, we need to exploit the details of rate optimization, including which indicator and adaptive algorithm to use and how to determine the final rate.

### 4.2. Rate-Optimization Algorithm

A typical backscatter network consists of one reader and one or more nodes. Accordingly, the link from reader to node is called the downlink, and the link from node to reader is called the uplink. An RA algorithm is the adaptive change of the data rate of these links. There are many differences between the downlink and the uplink, due to the mismatch in reader and node capabilities.

**Downlink**: The downlink adopts a simple PIE encoding, and its rate is determined by Tari, in the preamble, which is sent from the reader to the node. For Tari values, there are three options: 6.25 µs, 12.5 µs, and 25 µs, corresponding to the maximum downlink rates, 160 kbps, 80, kbps and 40 kbps [8].

**Figure 6:**
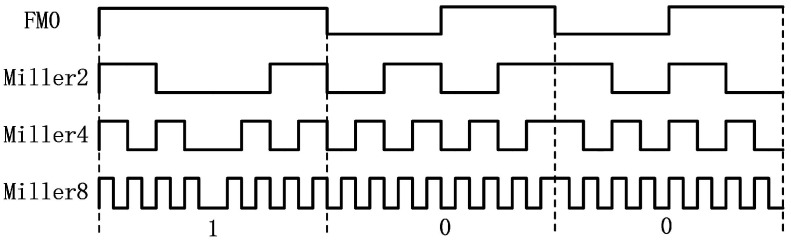
Different encoding methods at the same data rate.

**Uplink**: FM0 and Miller encoding are adopted in the uplink. The uplink rate is determined by the backscatter link frequency (BLF) and encoding method:

(4) rate=BLF/M

where BLF is equal to DR divided by TRcal, which are both determined by the reader. *M* is the number of subcarrier cycles. *M* = 1, 2, 3, and 4, whichcorrespond to modulated subcarriers FM0, Miller2, Miller4, and Miller8, respectively. Their corresponding waveforms are shown in Figure 6. With the same data rate, the bigger *M* is, a bit is represented by more code elements, and the anti-interference ability of the link is stronger, and vice versa.

Since Tari is not adjustable in an Impinj reader, we just optimize the rate of the uplink. In China, an Impinj speedway reader predefined five standard modes for the backward uplink, which is jointly determined by the encoding scheme and baud rate, as shown in Table 1. Like the previous works, we adopt two link indicators to describe the channel quality: (a) the RSSI value, for each response from a node, and (b) the cumulative packet-loss rate, for each dwell time interval. Given these two indicators, we can build a classifier to explore the inner mechanisms that influence the transmission and predict the most adequate rate of each effective node.

**Figure 7:**
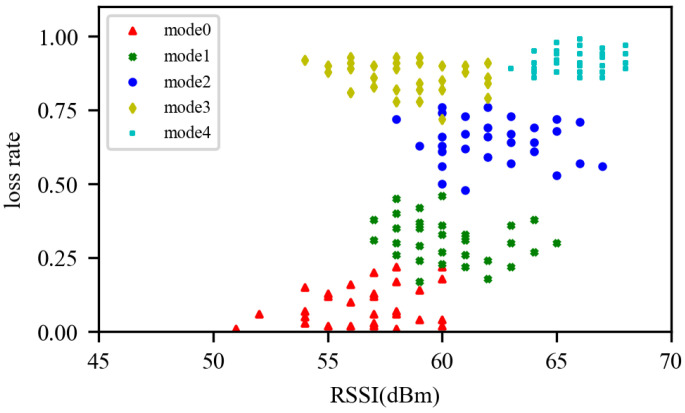
Optimal rate map.

The empirically measured optimal rate mode and their corresponding RSSI and loss rate is shown in Figure 7. Different colors on the map represent different rate modes. Obviously, these points gather together into clusters and can be divided into several categories. For each node, a classifier is needed to predict the optimal pattern for the next cycle. When the reader determines the rate, the requirement of all nodes need to be taken into account. To achieve this function, we adopt the well-known random forest algorithm, which takes a decision tree as the sub-classifier and integrates its results to get the final decision.

In order to implement the algorithm, we built a CART classification tree [20] for each node, to help to realize fast calculation and high real-time prediction. Each sub-classifier is a two-phase process, including a learning phase and a prediction phase. In the learning phase, we aim to build the classifier tree, by collecting channel indicators in the current environments, and label them with the corresponding optimal data rates. In the prediction phase, we obtain the optimal data rate of each node, by inputting the current channel indicators to the tree.

Assume that the size of the training set is *W*. $V_{ij}$ represents the *j*-th eigenvalue of the *i*-th sample. The steps of building a classification tree, using the CART algorithm, are as follows:

(1) Select an eigenvalue to be the criteria of each classification. The rule of the CART algorithm is to improve the purity of the sample set by finding the best eigenvalue, with a Gini coefficient that is the smallest. The Gini coefficient is defined as:

(5) $$\mathrm{gini}\left(a\right)=1-\sum_{k=1}^{K}p_{k}^{2}.$$

where *A* represents the target sample set, *K* represents the number of categories, and $p_{k}$ represents the proportion of the *k*-th category. We use $V_{best}$ to represent the best eigenvalue among all $V_{ij}$s. Accordingly, the sample set is divided into two categories, according to $V_{ij}\leq V_{best}$ and $V_{ij}>V_{best}$. In this paper, $V_{i1}$ and $V_{i2}$ (0 < *i* < *W*) represent the RSSI and loss rate of the i-th adaptation cycle, respectively.

(2) The obtained sets are further divided according to step (1), by selecting the eigenvalue again.

(3) Repeat steps (1) and (2), until the Gini coefficient in all leaf nodes is equal to 0.

(4) Input the current indicator into the decision tree and get the optimal rate mode. The current indicator will be added to the training set, for the next prediction, so the real performance of the model is guaranteed. In order to decide a fair public rate, the strategy of ensemble learning is applied to the random forest algorithm.

It is, generally, believed that the ensemble strategies can improve the generalization ability of the model [21], since the classification decision is made by a collection of base classifiers. In the case that the base classifiers show good diversity, the integrated approach can better deal with the bias–variance dilemma than the single model. For the sake of fairness, the initial ensemble strategy is the relative majority voting method, in which the largest number of predicted results of the base classifier is the final classification category. However, in order to improve the overall throughput of the whole system, we decide to give weighted voting to the rates of *N* nodes. According to the method, the votes of each base classifier are multiplied by a weight, and the weighted votes of each category are, finally, summed. Finally, the category corresponding to the largest value is chosen as the final optimal rate mode. The key to the performance of this algorithm is how to decide the weight. In RAEN, we assign the weights of the fast rate with large number, while the weights of the slow rate are assigned with a small number. The weight value is calculated by:

(6) $$w_{i}=\frac{{\mathrm{bitrate}}_{i}}{\sum_{i=1}^{l}{\mathrm{bitrate}}_{i}}.$$

where ${\mathrm{bitrate}}_{i}$ is the bitrate of the *i*-th mode, and *l* is the number of modes. In this paper, *l* is equal to 5. By weighted voting, higher rates are more likely to be selected, so the overall throughput of the network can be improved. Besides, the transmission of nodes with a low transmission rate are guaranteed.

## 5. Implementation and Evaluation

In this section, we present the implementation issues and evaluate the performance of each part.

### 5.1. Experiment Details

**Hardware**: We use an Impinj commercial reader and multiple passive RFID tags to compose a backscatter network. The commercial reader used is the Impinj Speedway R420, which is connected to the Laird circularly polarized directional antenna, S9028PCL. The reader can connect up to four antennas at the same time, and the antenna gain is 9 dBi [22]. The used tag is AZ-9662, with an Alien H3 chip.

**Software**: The data is collected by Octane SDK, based on Low-Level Reader Protocol (LLRP) [23], provided by EPC Global. LLRP is a standard, asymmetric, and binary communication protocol, which allows a client to control the configuration of reader before working. Based on LLRP, Octane SDK is developed to control the full functionality of Gen2 readers, so as to enable an easy software-development experience. In China, LLRP, by default, set the channel to hop between 16 frequencies, and the channel dwell time is fixed at 2 s [23]. As the existence of frequency diversity caused by frequency selective fading, channel qualities vary from channel to channel, which will result in inaccurate estimation of indicators and, finally, affect the effect of rate adaptation. In this paper, since we only focus on the optimization of rate, channel 1 is fixed, to exclude the effect of channel-frequency hopping.

### 5.2. Effective Nodes Extraction

Octane SDK allows us to access the phase information of nodes and to select nodes flexibly. In this part, we did experiments in an empty office, with an Impinj reader and serval RFID tags being set up. Our object is to identify effective nodes, timely and accurately, even if the change is minimal. To build the identification model, the effective node is monitored by the reader for 20 s. The trace is used as the input, to build the Gaussian model and to test for accuracy rates. As mentioned earlier, the effective nodes are moving or under a dynamic environment. To evaluate the first case, we placed a tag 30 cm away from the reader and moved it to a random direction, with a displacement ranging from 1 cm to 6 cm. To evaluate the second case, we made a pendulum, by tying the tag to a string, and the swing speed of tag is adjustable to low, medium, and high. Each displacement setting is repeated 100 times, and the true positive rate (TPR) is used to evaluate the identification accuracy.

**Figure 8:**
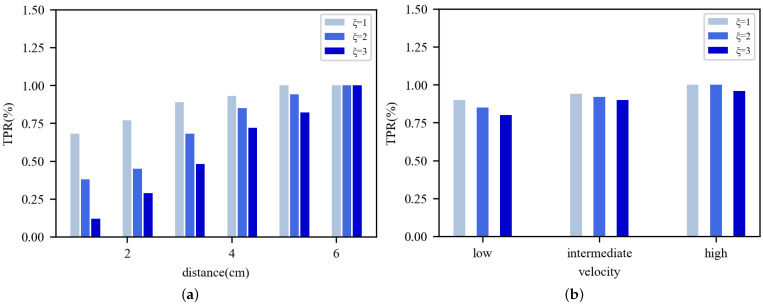
Accuracy when identifying effective nodes by different thresholds. (**a**) Moving. (**b**) Dynamic environments.

Figure 8a presents the accuracy results of identifying effective nodes that are moving, where different threshold values, such as ξ = 1, ξ = 2, and ξ = 3, are compared. According to the Gaussian distribution, ξ = 1, 2, and 3, respectively, represent confidence levels of 88.27%, 95.45%, and 99.73%. The TPR of different threshold values is between 80% and 100%. The figure shows that we can successfully detect most of the movement events using the Gaussian model, when the tag is moved away from 1 cm to 6 cm. When the tag is moved away to 6 cm, the method can achieve a 100% success rate, under all threshold values. What’s more, an increase in the threshold results in a decrease in TPR. So, we set the threshold to 2, as a compromise. The similar conclusion can, also, be drawn in Figure 8b, where the effective nodes are under dynamic environments. On the whole, effective nodes identification, which is based on the Gaussian model, can be applied to actual environments and own a high accuracy.

### 5.3. Rate Optimization

#### 5.3.1. Determination of *M*

**Figure 9:**
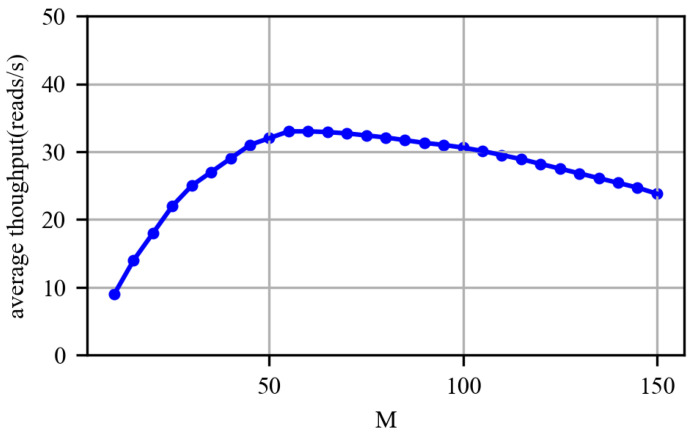
The impact of period length on throughput.

As mentioned above, *M* is the threshold value of the number of inventory cycles. In order to find the most appropriate *M*, we randomly placed 10 tags 30 cm away from the reader within the beam, and measured their throughput for 10 min, with three people hanging around to create a dynamic environment. We then changed the value of *M* from 10 to 100, to repeat the experiment. The nodes’s average throughput under different periods are shown in Figure 9. The average throughput of the nodes, first, increases and, then, decreases, with the increase in *M*. That is because the reader will spend tens of milliseconds to switch the rate, so switching too frequently is not efficient. In addition, as *M* increases, it is more and more difficult to keep up with the changes in the environment. In our experimental scenario, the average throughput of the nodes is maximized, when *M* is equal to 55. However, the value of *M* varies with the number of tags and the environment. In the following experimental evaluation, *M* is not a fixed value, but a quantity that is measured in real time, upon a specific case.

#### 5.3.2. Rate Optimization Efficiency

**Figure 10:**
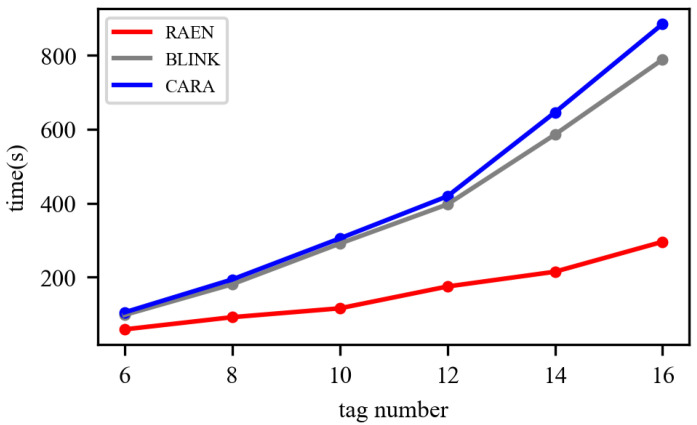
Efficiency of the trigger mechanism.

In order to prove that our trigger mechanism is more efficient than the previous detection-based method, we did an experiment to observe the consumed prediction time, by changing the number of tags from 6 to 16. The tags are, also, placed at 30 cm away from the reader, within the beam. As shown in Figure 10, we compare the time cost of RAEN against Blink and CARA. For fairness, the extraction part of RAEN is removed. Obviously, the time cost of Blink and CARA grows quadratically with the number of nodes, while that of RAEN increases linearly. For example, when there are 16 tags, the probing costs of Blink and CARA are 895 ms and 820 ms, corresponding to 2.9× and 2.6× more than that of RAEN.

#### 5.3.3. Rate Optimization Accuracy

**Figure 11:**
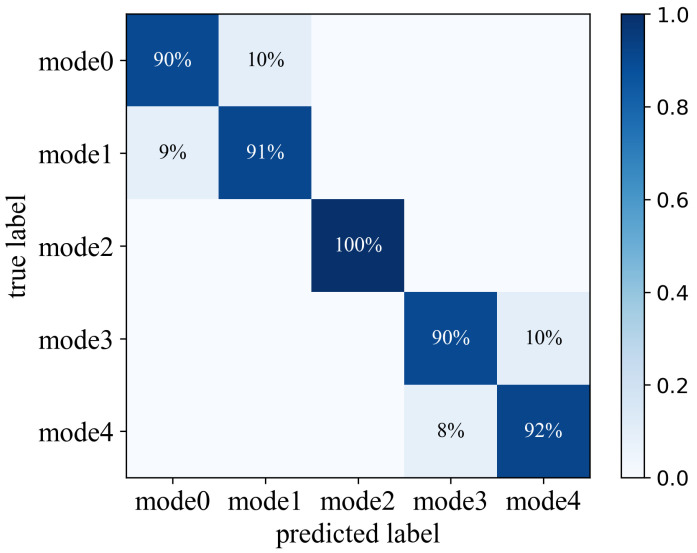
Confusion matrix of rate adaption over different modes.

LLRP provides us five kinds of data rates for the backward uplink. The decision-tree classifier and weighted-voting strategy help us choose the optimal rate of the current transmission environment. To verify the accuracy of our rate optimization method, we place 20 tags at a random location, relative to the reader; with their optimal rate and the corresponding RSSI, packet loss been measured. The results are shown in Figure 11, where we build a confusion matrix, which includes all rate modes, to show the accuracy. In the confusion matrix, the column and the row, respectively, stand for the predicted and the actual rate mode of a sample tag. We achieve more than a 90% rate of prediction success, which shows the accuracy of our scheme against time.

### 5.4. Overall Evaluation

**Figure 12:**
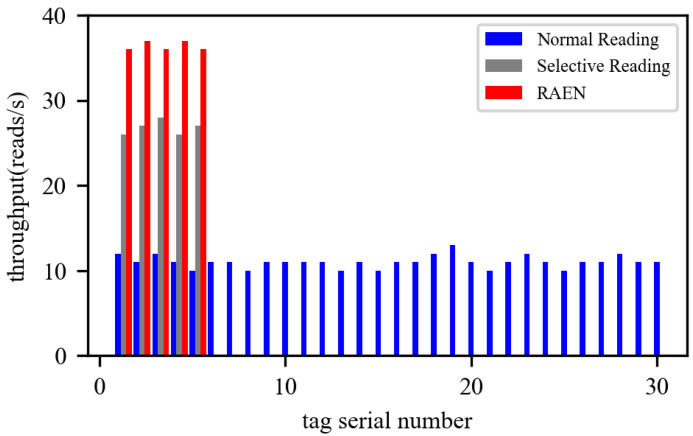
The improvement effect on effective node throughput.

Finally, we evaluate the overall performance of RAEN based on a trace-driven simulation. We, first, evaluate the overall performance from the perspective of each node. In Figure 12, we randomly place 30 tags on a testbed, which is perpendicular to the normal direction of the antenna. The testbed is located 30 cm away from the antenna and has a size of 2 m × 3 m. In order to measure the improvement effect of RAEN on the throughput of effective nodes, we conduct the effectiveness by seting 1/6 of the tags to move. Each mobile tag has a fixed trajectory and is carried by a car, with a velocitiy that is 0.3 m/s. We compare RAEN with a normal reading, where all nodes are treated no differently, and selective reading, where the effective nodes are extracted, but the rate is not optimized. The throughput of each tag is measured 20 times, and Figure 12 shows the average value of the measurement results. Take tag1 as an example; its throughput is 12 reads/s, in the solution of normal reading. However, if selective reading is applied, the throughput of tag1 will increase to 2.2× (i.e., from 12 reads/s to 26 reads/s). Meanwhile, the throughput of invalid nodes drops to zero. What’s more, when RAEN is applied, the throughput of tag1 is further improved to 3× (i.e., from 12 reads/s to 36 reads/s). The throughput of other effective nodes has a similar improvement. We, then, vary the initial location and velocity of the nodes and similar conclusions can be drawn.

**Figure 13:**
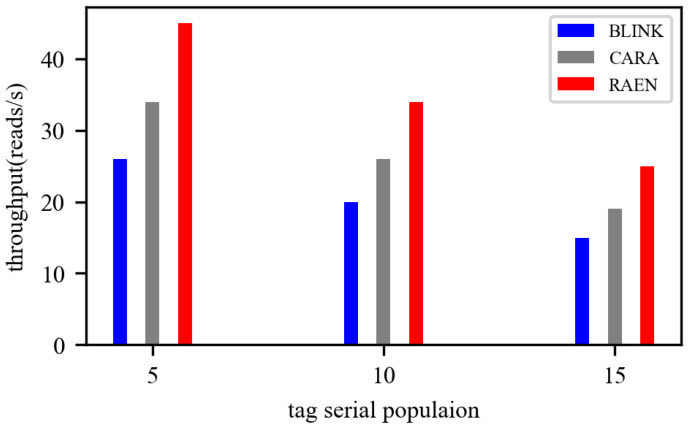
Overall throughput comparison for tags.

We, now, evaluate the overall performance of the whole framework and compare it with traditional rate-adaption methods, such as Blink and CARA. Since RAEN merely give priority to effective nodes, we only consider the case where all nodes are effective, by changing their environmemnt. We conduct over 50 tests and fix the initial distance between reader and tags at 0.5 m. The population of tags varies from 5 to 15. One volunteer walks around the nodes, to create a dynamic environmemnt. The results of the different populations of tags are shown in Figure 13. We find that the throughput of RAEN is approximately 1.3× and 1.7× better than CARA and Blink, on average. The same trend can be observed when the number of tags increases. As expected, all schemes degrade with the increasing number of nodes because more coordination time is needed.

## 6. Discussion

Since RAEN takes node effectiveness and rate optimization both into consideration, its performance is better than only extracting nodes (see Figure 12) or only optimizing the rate (see Figure 13). The two modules complement each other, and the specific results are discussed below.

When identifying effective nodes (see Figure 2), it is easy to understand that moving will cause an irregular distribution of phase value, since the distance between the reader and tag is changing. As for phase-distribution jitter, caused by environmental change, the root cause of this phenomenon is the multipath effect [24]. The relationship between the accuracy of effective nodes identification and the threshold values (see Figure 8) shows that the method can identify most situations of effective nodes. It is, mainly, due to the phase being extremely sensitive to changes in the environment. What’s more, we found a continuous increase in TPR over the threshold values. Similar conclusions can be drawn from [25].

The efficiency of the rate-optimization module shows its superiority over traditional methods (see Figure 10). This is, primarily, because the detection-based method needs to detect the mobility of the channel via additional probes, while our periodic method leaves this step out. Besides, the high real-time performance random forest algorithm, also, saves the cost. The accuracy of rate optimization, also, shows excellent performance (see Figure 11). However, the boundary errors in the empirical rate map make the rate-adaption optimization degrade, but the similar performance of the boundary points keeps the overall throughput from being affected too much. These findings, also, adhere to the results reported by [5,6,8], for the same study area.

## 7. Conclusions

In this paper, we present RAEN to improve the throughput of a backscatter network, by allowing effective nodes to transmit exclusively in an appropriate data rate. RAEN adopts the Gaussian Model to identify effective nodes. Then, an effective nodes-selection method is leveraged, to achieve batch selection of effective nodes. After that, a random forest classifier is used to predict the most adequate rate mode. Comprehensive experiments demonstrate that RAEN can improvesthe throughput of effective nodes by 3×, when 1/6 of nodes are effective, compared with traditional methods. The RAEN scheme is an obvious improvement over normal reading and existing schemes, and will provide many new opportunities for application scenarios.

## Figures and Tables

**Table 1:** Standard modes of Impinj speedway reader.

Mode	Name	Encoding/Baud Rate	Bitrate (kbps)
0	Max Throughput	FM0/640	640
1	Hybrid	M2/320	160
2	Dense Reader M4	M4/320	80
3	Dense Reader M8	M8/320	40
4	Max Miller (M4)	M4/640	160

## Data Availability

Not applicable.
